# Quantitative Assessment of Salivary Gland Parenchymal Vascularization Using Power Doppler Ultrasound and Superb Microvascular Imaging: A Potential Tool in the Diagnosis of Sjögren’s Syndrome

**DOI:** 10.4274/balkanmedj.galenos.2020.2019.11.91

**Published:** 2020-06-01

**Authors:** Fethi Emre Ustabaşıoğlu, Selçuk Korkmaz, Ufuk İlgen, Serdar Solak, Osman Kula, Sezin Turan, Hakan Emmüngil

**Affiliations:** 1 Department of Radiology, Trakya University School of Medicine, Edirne, Turkey; 2 Department of Biostatistics and Medical Informatics, Trakya University School of Medicine, Edirne, Turkey; 3 Department of Rheumatology, Trakya University School of Medicine, Edirne, Turkey

**Keywords:** Power Doppler ultrasound, primary Sjögren’s syndrome, salivary glands, superb microvascular imaging, ultrasonography

## Abstract

**Background:**

Primary Sjögren’s syndrome is a chronic inflammatory autoimmune disease. Minor salivary gland biopsy is the gold standard for the diagnosis of primary Sjögren’s syndrome. Superb microvascular imaging, power Doppler ultrasound, and color Doppler of the salivary glands represent non-invasive, non-irradiating modality for evaluating the vascularity of the salivary glands in the diagnosis and follow-up of primary Sjögren’s syndrome.

**Aims:**

To evaluate the efficacy of superb microvascular imaging and vascularity index in salivary glands for the sonographic diagnosis of primary Sjögren’s syndrome.

**Study Design:**

Prospective case-control study.

**Methods:**

Twenty participants with primary Sjögren’s syndrome and 20 healthy subjects were included in the study. Both parotid glands and submandibular glands were evaluated by superb microvascular imaging, power Doppler ultrasound, and color Doppler. The diagnostic accuracy of superb microvascular imaging was compared using these techniques.

**Results:**

In the patient group, the vascularity index values of superb microvascular imaging in parotid glands and submandibular glands were 3.5±1.66, 5.06±1.94, respectively. While the same values were 1.0±0.98 and 2.44±1.34 in the control group (p≤0.001). In the patient group, the vascularity index values of power Doppler ultrasound in parotid glands and submandibular glands were 1.3±1.20 and 2.59±1.82, respectively. While the same values were 0.3±0.32 and 0.85±0.68 in the control group (p≤0.001). The superb microvascular imaging vascularity index cut-off value for the diagnosis of primary Sjögren’s syndrome in parotid glands that maximizes the accuracy was 1.85 (area under the curve: 0.906; 95% confidence interval: 0.844, 0.968), and its sensitivity and specificity were 87.5% and 72.5%, respectively. While the superb microvascular imaging vascularity index cut-off value for the diagnosis of primary Sjögren’s syndrome in submandibular gland that maximizes the accuracy was 3.35 (area under the curve: 0.873; 95% confidence interval: 0.800, 0.946), its sensitivity and specificity were 82.5% and 70%, respectively.

**Conclusion:**

Superb microvascular imaging with high reproducibility of the vascularity index has a higher sensitivity and specificity than the power Doppler ultrasound in the diagnosis of primary Sjögren’s syndrome. It can be a noninvasive technique in the diagnosis of primary Sjögren’s syndrome when used with clinical, laboratory and other imaging methods.

Primary Sjögren’s syndrome (pSS) is a systemic disease characterized by xerostomia and keratoconjunctivitis sicca in which the autoimmune response of cellular and humoral mechanisms affects the salivary glands (1). In the parenchyma of the salivary gland, pathologic damage to the acinis, secondary to lymphocyte infiltration and fibrosis, are seen (2). There should be no other rheumatologic disease for the diagnosis of pSS. In secondary SS, there are diseases such as systemic lupus erythematosus and rheumatoid arthritis. When making a diagnosis of pSS, tests such as sialoscintigraphy, and serologic, and both pathologic and clinical findings are used (3,4,5).

Salivary gland sonography is a non-invasive method that does not involve ionizing radiation and has a major significance in the diagnosis of pSS (6,7). Major sonographic findings in patients with pSS are heterogeneous parenchyma with multiple hypoechoic areas and reticular patterns because of hyperechoic stripes (8). Hypoechoic areas usually have a radius of 2-5 mm and are caused by lymphocytic infiltration, whereas echogenic stripes are caused by fibrosis and fatty infiltration (9,10). Recently, salivary gland sonography has been proposed as a promising, highly specific, and non-invasive modality for the diagnosis of pSS even in the early clinical stages (11). Sonographic scoring systems are underway (12), and parotid ultrasonography was mentioned as an upcoming diagnostic test at the 2016 American College of Rheumatology/European League Against Rheumatism (ACR/EULAR) pSS classification consensus even though it is still not included in the current classification criteria (13). Color Doppler (CD) and power Doppler ultrasound (PDUS) are other important techniques used in pSS. Superb microvascular imaging (SMI), on the other hand, is a more sensitive vessel imaging modality than these two methods and can show smaller vessels than CD and PDUS imaging (14). In a recent study (15) performed on parotid glands, SMI values were significantly higher than PDUS and CD values in healthy children and adolescents.

Our study is the first to measure the vascularity of the salivary gland parenchyma in patients with pSS using SMI. We aimed to evaluate the salivary gland parenchyma using grayscale ultrasound (US) and degree of vascularity using SMI and PDUS in patients with pSS.

## MATERIALS AND METHODS

Our study was conducted at Trakya University Radiology Department between March and May 2019. The local ethics committee of Trakya University School of Medicine approved the study protocol (no: TUTF-BAEK 2019/109 date: 11.03.2019). All patients who participated in the study gave informed consent.

### Patient population

Our study was designed prospectively, and 20 patients (20 women) with pSS and 20 healthy controls (20 women) were evaluated. Consecutive patients with pSS from the Rheumatology Department who met the 2016 ACR/EULAR criteria and who had a focus score of at least one/4 mm2 on the labial salivary gland biopsy, were included in the study. The activity of the disease was assessed using the EULAR Sjögren’s syndrome disease activity index (16). Antinuclear antibody and autoantibodies against Ro (SS-A) and La (SS-B) were tested using an indirect immunofluorescence assay (Euroimmun, Lübeck, Germany).

Control subjects were chosen from volunteers of a similar age and sex who had no clinical signs or symptoms of pSS. Patients with any systemic disease or drug use that might affect salivary glands were excluded. Participants receiving antidepressant therapy, anticholinergic drugs, or drugs that affect the salivary glands, and those who were diagnosed as having sarcoidosis, hepatitis C infection, pre-existing lymphoma, or who had a history of salivary gland surgery and head and neck radiation were also excluded.

parotid glands and submandibular glands of patients and controls were examined using US, PDUS, and SMI, and all the measurements were statistically analyzed.

### Sonography technique

Two radiologists (FEU, SS) with 9 years’ radiology experience who were blinded to all clinical information of the patients separately performed US, PDUS, and SMI on all participants (20 patients and 20 controls) on the same day. Both examiners were blinded to each other’s examination results and the patients’ symptoms. Both the patient and the control group were evaluated twice for interobserver variability analysis. Before initiation of the study, eight healthy control subjects were selected for PDUS and SMI technique standardization. These examinations were not included in the statistical analysis.

Parotid glands and submandibular glands were examined using an Aplio 500 Platinum ultrasound device (Canon Medical Systems, Japan) with a 5-14 MHz linear probe. The US of the salivary glands was performed while participants were in the supine position with their necks were extended, and their heads were turned to the opposite side. After this, patients’ heads were tilted back maximally to access the submandibular area.

Parotid glands were evaluated in the retromandibular fossa, anterior to the ear and sternocleidomastoid muscle, and submandibular glands were evaluated in the posterior part of the submandibular triangle. The thyroid was also scanned for comparison with the salivary glands for the evaluation of parenchymal echogenicity.

Parenchymal echogenicity, inhomogeneity, presence of hypoechoic areas, and hyperechoic foci in the salivary glands were used as US parameters.

### Power Doppler ultrasound and superb microvascular imaging technique

**Figure 1:**
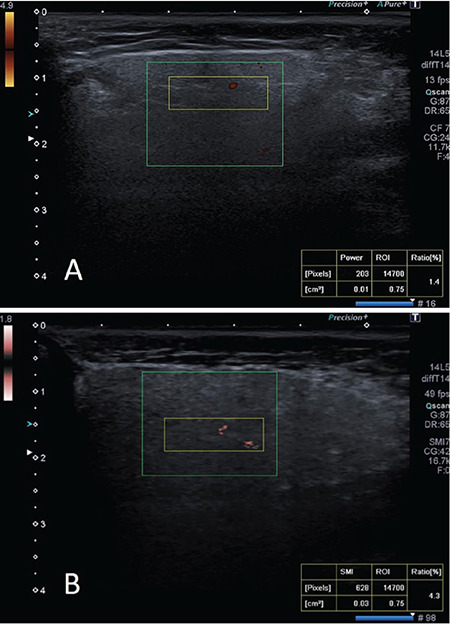
(A) Power Doppler ultrasound and (B) superb microvascular imaging, examination of parotid gland in a 37-year-old patient with primary Sjögren syndrome. An superb microvascular imaging of the patient (B) shows a substantially higher amount of blood fl ow (VI, 4.3%) compared with the power Doppler ultrasound image of the patient (A; VI, 1.4%).

An 870 to 966 Hz pulse repetition frequency and 10-15 frame rate were used in PDUS measurements. The SMI examination was performed on the same area as the US with standardized parameters of the ultrasound device, without compression. A colored box with a 3×2 cm fixed window was placed and adjusted to examine the gland. Next, the observer captured the image, positioned the standardized (15×5 mm) region of interest at the center of the gland, and automatically calculated the vascularity index. The vascularity index represents the percentage of color pixels in the total grayscale pixels in a defined region of interest. The same measurement protocol was used for both PDUS and SMI evaluations. The normal large vessels visible within the salivary glands (external carotid artery and retromandibular vein in the parotid gland and facial artery and vein in the submandibular gland) were excluded from the PDUS and SMI vascularity index scores. All images were transferred electronically to a picture archiving system (Sectra PACS Linköping-SWEDEN). Figure 1 shows the SMI and PDUS evaluations of a patient.

### Statistical analysis

The Shapiro-Wilk test was used to analyze the normality of data distribution. Quantitative variables were expressed as the mean and standard deviation or median and interquartile range based on their distribution. Categorical variables were expressed as frequencies and percentages. The group comparisons were performed using a Student’s t-test if data follow a normal distribution, whereas a Mann-Whitney U test was used when the data distribution was not normal. Fisher’s exact test was used to assess the relationships between categorical variables. The Bonferroni adjustment of p-values was used for multiple testing. The receiver operating characteristic (ROC) curve analysis and Youden index were used to detect cut-off values for PDUS and SMI. Interobserver agreement in the measurements was calculated using intraclass correlation coefficients. A 95% confidence interval (CI) was constructed for each intraclass correlation coefficient. A p-value of less than 0.05 considered statistically significant. All statistical analyses were conducted using SPSS version 16 (Chicago, IL).

## RESULTS

Both parotid glands and submandibular glands of 20 patients with pSS and 20 control subjects were evaluated. There was no statistically significant difference between the mean ages of the patient (56.75±14.07 years) and control (55.55±13.67 years) groups (p=0.786). All patients included in the study were female. In US imaging, the difference between patients with PSS and the control group was statistically significant for heterogeneity (p<0.001), presence of hypoechoic areas (p<0.001), and hyperechoic foci (p=0.02). These results are summarized in Table 1.

The PDUS and SMI vascularity index values of both parotid glands and submandibular glands of the patient and control groups are presented in Table 2. The vascularity index values of SMI in both parotid glands and submandibular glands were remarkably higher in the patient group than in the control group (p<0.001). Similarly, the vascularity index values of PDUS in both parotid glands and submandibular glands were statistically higher in the patient group compared with the control group (p<0.001).

The efficacy and specificity of SMI and PDUS in our study population were investigated using ROC curves. ROC curves were calculated and were compared with the obtained vascularity index values. The vascularity index values acquired using the SMI method were significantly higher than the PDUS vascularity index values (p<0.001).

**Figure 2:**
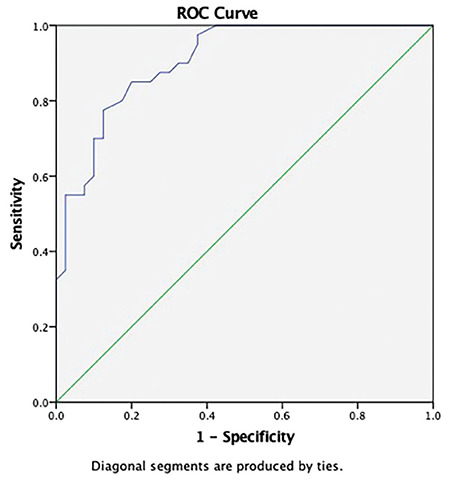
Superb microvascular imaging significantly discriminates primary Sjögren syndrome in the parotid gland from healthy subjects according to the ROC curve analysis (AUC:0.906, 95% CI: 0.844-0.968).

**Figure 3:**
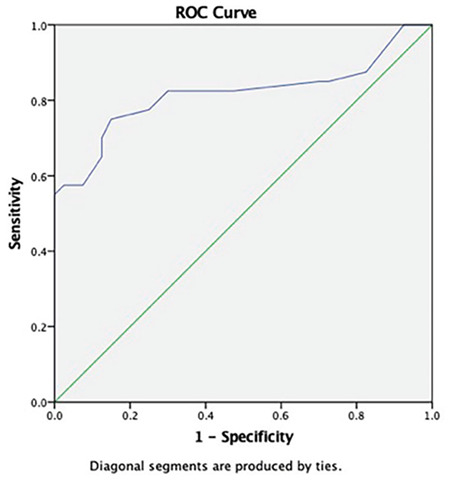
Power Doppler ultrasound significantly discriminates primary Sjögren syndrome in the parotid gland from healthy subjects according to the ROC curve analysis (AUC:0.817, 95% CI: 0.716-0.918).

In both the patient and control groups, values of vascularity index with PDUS or SMI were not significantly different between the right and left side. Figures 2 and 3 show the ROC curve that was drawn based on the SMI and PDUS vascularity index values for the diagnosis of pSS.

The SMI vascularity index cut-off value for the diagnosis of pSS in parotid gland that maximized the Youden index was 1.85 (Area under the curve (AUC): 0.906; 95% confidence interval (CI): 0.844-0.968), and its sensitivity and specificity were 87.5% and 72.5%, respectively. The SMI vascularity index cut-off value for the diagnosis of pSS in submandibular gland that maximized the Youden index was 3.35 (AUC: 0.873; 95% CI: 0.800-0.946), and its sensitivity and specificity were 82.5% and 70%, respectively.

The PDUS vascularity index cut-off value for the diagnosis of pSS in parotid gland that maximized the accuracy was 0.55 (AUC: 0.817; 95% CI: 0.716-0.918), and its sensitivity and specificity were 82.5% and 70%, respectively. The PDUS vascularity index cut-off value for determining the diagnosis of pSS in submandibular gland that maximized the accuracy was 1.45 (AUC: 0.862; 95% CI: 0.716-0.918), and its sensitivity and specificity were 77.5% and 67.5%, respectively.

Interobserver variability results are shown in Table 3. There was excellent agreement between the two blinded observers for both the patient and control groups in SMI and PDUS vascularity index measurements (intraclass correlation coefficient and 95% CI: 0.994 (0.991-0.996) for SMI vascularity index and 0.965 (0.945-0.977) for PDUS vascularity index).

## DISCUSSION

Salivary gland inflammation is a highly frequent clinical presentation in patients with pSS and salivary gland destruction that can be correlated with disease activity (17). In the diagnosis of pSS, histopathologic confirmation of the disease by minor salivary gland biopsy remains the gold standard. However, in recent years, salivary gland sonography has gained an important place in diagnosis (18,19).

Both CD and PDUS can show increased vascularity caused by inflammation. Thus, they can be used in the diagnosis of pSS. PDUS is an integrated power spectrum that depends on a low angle and has higher sensitivity in detecting low blood flow velocity. On the other hand, the CD is based on the mean Doppler frequency shift (20,21). According to the developed filtering techniques in SMI, background noises are eliminated, and small, slow velocity vascular structures can be imaged successfully. Compared with standard PDUS, SMI has higher sensitivity and resolution (22). Indeed, in the present study, SMI was found to be superior to PDUS in identifying salivary gland vascularity.

In a study in which SMI properties of parotid glands were investigated, Caliskan et al. (15) studied the vascularization of parotid gland in healthy children using SMI and PDUS. However, in this study, no quantitative method such as vascularity index was used, and they only compared the numbers of vascular spots numbers as a semi-quantitative method. Shimizu et al. (23) stated that vascularization in patients with pSS was increased by finding 4-6 vascular spots in parotid gland using the CD. However, a conventional CD is not capable of detecting very slow blood flow. SMI can be much more effective than CD and even PDUS in the diagnosis of pSS since it can detect slowly moving blood.

Our study has some limitations. The main limitation of our study is the small patient population that comprised only female patients. Second, the patients were examined using ultrasonography, and salivary glands were evaluated regarding gland heterogeneity, the presence of hypoechoic areas, presence of hyperechoic foci, and clearance of submandibular gland posterior borders. However, the relationship between these parameters and the degree of SMI was not analyzed. Another limitation was the lack of elastographic evaluation. Though, the main goal of this study was to evaluate the degree of vascularity and emphasize the role of SMI, which is a new diagnostic tool.

In conclusion, our study showed that SMI has higher sensitivity and specificity than PDUS in the diagnosis of pSS. Quantitative SMI vascularity index values, which have high reproducibility, could become a useful non-invasive tool for the diagnosis of pSS along with clinical parameters, laboratory findings, and other imaging modalities, such as US and PDUS.

## Figures and Tables

**Table 1:**
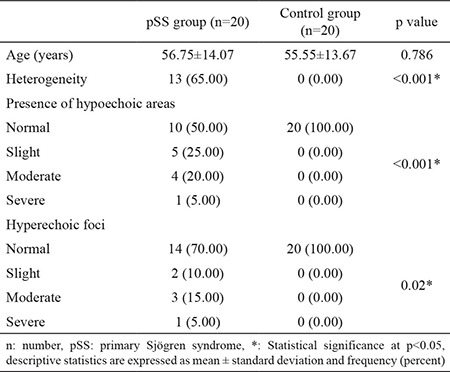
General characteristics and sonographic findings of the study population

**Table 2:**
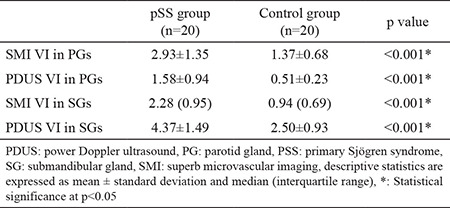
Comparison between patients with primary Sjögren syndrome and the control group based on superb microvascular imaging and power Doppler ultrasound, VI values for both parotid glands and submandibular glands

**Table 3:**
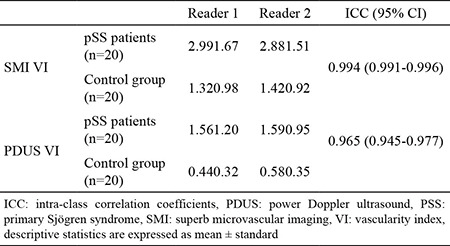
Interobserver variability for superb microvascular imaging VI and power Doppler ultrasound VI values of parotid glands
